# Vaccine-Derived Polioviruses, Central African Republic, 2019

**DOI:** 10.3201/eid2702.203173

**Published:** 2021-02

**Authors:** Marie-Line Joffret, Joël Wilfried Doté, Nicksy Gumede, Marco Vignuzzi, Maël Bessaud, Ionela Gouandjika-Vasilache

**Affiliations:** Institut Pasteur, Paris, France (M.-L. Joffret, M. Vignuzzi, M. Bessaud);; Institut Pasteur, Bangui, Central African Republic (J.W. Doté, I. Gouandjika-Vasilache);; World Health Organization African Region Office, Brazzaville, Congo (N. Gumede)

**Keywords:** Poliovirus, oral polio vaccine, vaccine-derived poliovirus, poliomyelitis, eradication, viruses, Central African Republic

## Abstract

Since May 2019, the Central African Republic has experienced a poliomyelitis outbreak caused by type 2 vaccine-derived polioviruses (VDPV-2s). The outbreak affected Bangui, the capital city, and 10 districts across the country. The outbreak resulted from several independent emergence events of VDPV-2s featuring recombinant genomes with complex mosaic genomes. The low number of mutations (<20) in the viral capsid protein 1–encoding region compared with the vaccine strain suggests that VDPV-2 had been circulating for a relatively short time (probably <3 years) before being isolated. Environmental surveillance, which relies on a limited number of sampling sites in the Central African Republic and does not cover the whole country, failed to detect the circulation of VDPV-2s before some had induced poliomyelitis in children.

Poliomyelitis results from infection of the central nervous system by poliovirus, a picornavirus of the species *Enterovirus C* (*1*). The Global Polio Eradication Initiative (https://polioeradication.org) managed to eradicate wild poliovirus of 2 of the 3 serotypes and to contain virus of the third serotype in Pakistan and Afghanistan. The Initiative relies on 2 pillars: surveillance of poliovirus circulation and vaccination. Contrary to the inactivated polio vaccine, the oral polio vaccine (OPV) induces strong intestinal immunity that blocks transmission of poliovirus in subsequent infections (*2*). Consequently, OPV is currently the only tool capable of stopping poliovirus transmission. However, because attenuated strains of OPV replicate in the gut and are excreted in feces, low vaccine coverage enables circulation of these strains and loss of their attenuated phenotype through genetic drift (*3*,*4*). Since May 2019, the Central African Republic (CAR) has experienced a poliomyelitis outbreak caused by serotype 2 vaccine-derived polioviruses (VDPV-2s). To ascertain the origin of these VDPV-2s, we determined and analyzed their full-length genomic sequences.

## The Study

**Figure 1:**
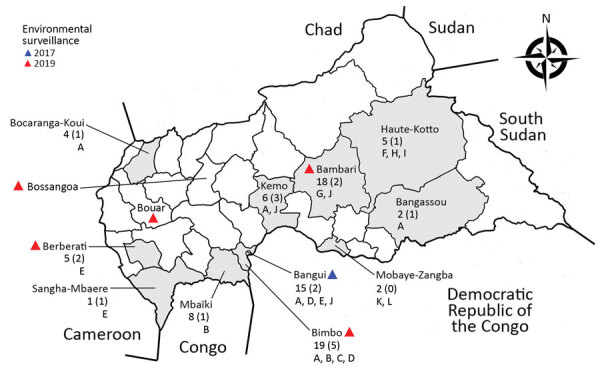
Central African Republic. Shading indicates districts where VDPV-2s were detected May–December 2019: triangles indicate districts where environmental surveillance has been implemented; numbers indicate total numbers of VDPVs; numbers in parentheses indicate number of confirmed poliomyelitis cases, letters A–L indicate VDPV lineages (based on the viral capsid protein 1–encoding region [Figure 2, panel A]). VDPV-2, type 2 vaccine-derived polioviruses.

During May–December 2019, using standardized procedures of the Global Polio Laboratory Network (https://polioeradication.org), we detected VDPV-2s in fecal samples of 19 children with acute flaccid paralysis (AFP). Positive samples came from 10 districts across the country, including Bangui, the capital city (Figure 1). In addition, we detected 49 VDPV-2s in fecal samples from healthy children living in the vicinity of the children with poliomyelitis; 17 were detected in environmental samples. In December 2017, routine environmental surveillance was implemented in CAR, restricted to 4 sampling sites in Bangui; 6 additional sites were gradually opened in 2019 (Figure 1). Compared with the vaccine Sabin-2 strain (reference strain), CAR VDPV-2s had 6–20 nt differences in the viral capsid protein 1 (VP1)–encoding region (903 nt), above the threshold used to discriminate VDPV-2s from Sabin-2 (>6 mutations within the VP1-encoding sequence). Given the evolutionary rate of this genomic region (≈10^−2^ nucleotide changes/site/year [*5*]), this range suggests that VDPVs had been circulating in CAR from a few months to a couple of years before detection.

**Figure 2:**
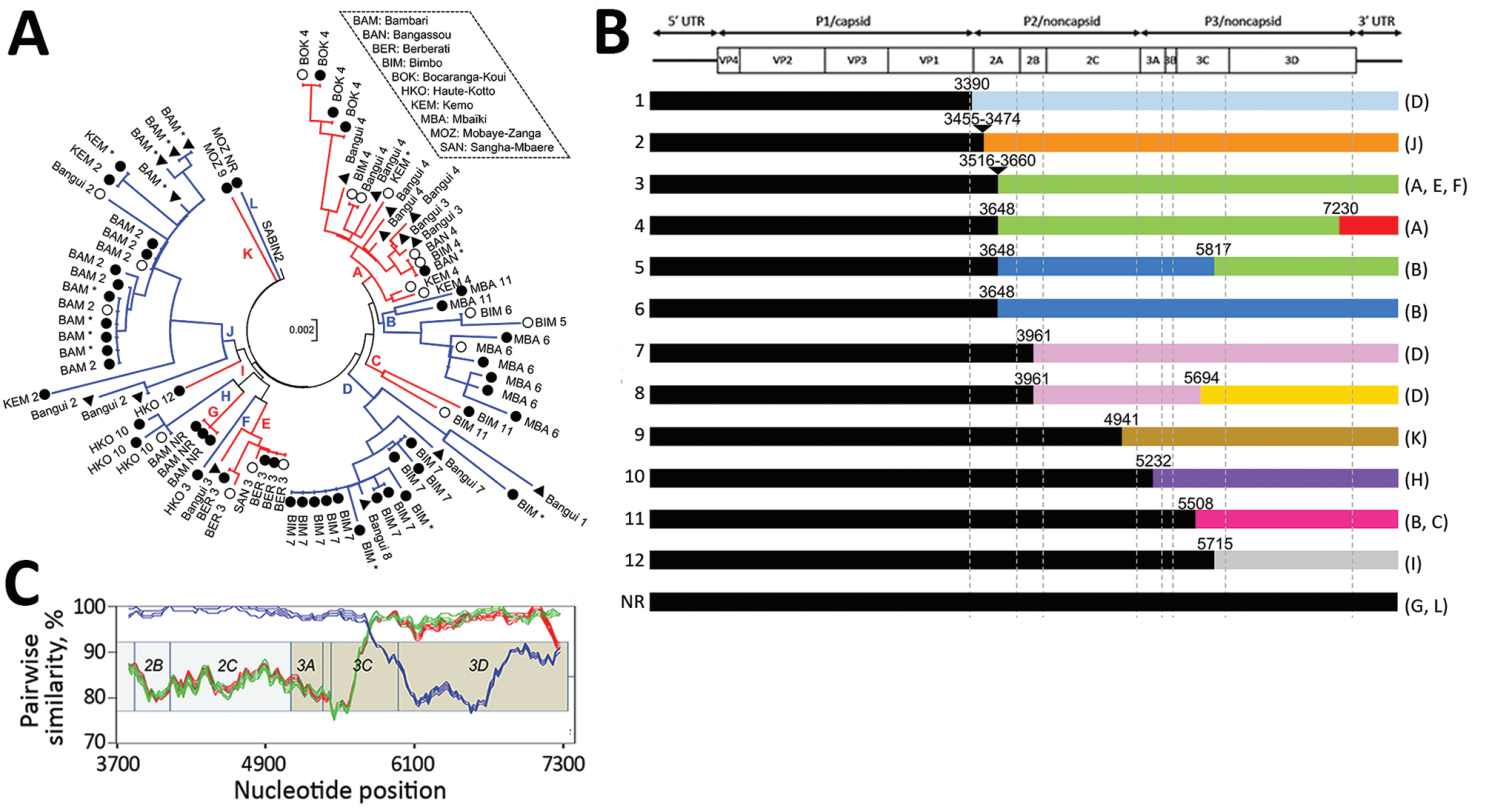
Molecular characterization of VDPV-2s isolated in the Central African Republic in 2019. A) Phylogram of the VP1-encoding sequence drawn by using the maximum-likelihood method based on the data-specific model. Alternating blue and red indicate evolutionary branches (A–L); open circles indicate sequences of VDPVs from patients with acute flaccid paralysis circles; closed circles indicate sequences of VDPVs from healthy children; black triangles indicate sequences of VDPVs from environmental samples. The district where the isolate was sampled and the recombinant pattern the isolate belongs to (patterns 1–12 or nonrecombinant [Figure 2, panel B]) are indicated; asterisks indicate isolates that have not been fully sequenced. Scale bar indicates nucleotide substitutions per site. B) Schematic representation of the genomic patterns of the VDPVs. Top row shows poliovirus genetic organization, with the main open reading frame flanked by the 5′ and 3′ untranslated regions (UTRs). Approximate locations of the recombination sites (1–12 on left) are shown. Sequences with different colors differ by <3%. Letters in parentheses on the right indicate the VP1 branches where each recombinant pattern can be found. NR, no recombination. C) Similarity plot drawn by comparing the sole genome of pattern 5 with genomes of patterns 3 (green), 4 (red), and 6 (blue) in the 3′ half of the genome. Sliding window width, 200 nt; step distance, 20 nt. VDPV-2s, type 2 vaccine-derived polioviruses; VP1, viral capsid protein 1.

Phylogenetic analysis based on VP1-encoding regions showed that the CAR VDPV-2s fell into different lineages (Figure 2, panel A; Appendix Figure). Although the low number of nucleotide differences in young VDPVs makes precise marking of the boundaries of phylogenetic clusters challenging, we identified >12 main branches in this phylogram (Figure 2, panel A, branches A–L), indicating the concomitant emergence of multiple VDPV lineages. Branches A and J gathered sequences of VDPVs sampled from districts located hundreds of kilometers apart (Figure 1), which suggests active circulation of these lineages in the country; by contrast, some lineages (F, I, K, L) were detected only 1 time. No isolates from patients with AFP were of lineages D, F, G, I, K, L; however, determining whether some AFP cases were missed or, alternately, whether surveillance managed to uncover VDPV lineages before they caused poliomyelitis, is not possible. Environmental surveillance is expected to detect poliovirus circulation before it causes the first poliomyelitis case, but the alert system is efficient only if the surveillance is dense enough to cover the entire country, a goal that is difficult to reach in CAR because of the political troubles.

Among the 70 CAR VDPV-2s for which genomes have been fully sequenced through gene walking and Sanger sequencing, only 4 (branches G and L, from healthy children) were free of recombination events and feature a global nucleotide divergence <1% compared with Sabin-2. The 66 other CAR VDPV-2 genomes comprised sequences derived from Sabin-2 and from other nonpolio enteroviruses in 12 recombinant patterns; polio/nonpolio breakpoints were within the 2A, 2B, 2C, 3A, 3C, and 3D-encoding regions (Figure 2, panel B; Appendix Figure). In the nonpolio region, the unique representative of recombinant pattern 5 (member of VP1 branch B) shared recent common ancestors through recombination with the genomes of patterns 3 and 6: it was closely related to genomes of pattern 6 from the 2A through the 3C genomic regions and to the genomes of pattern 3 downstream (Figure 2, panel C). Pattern 4 also shared a recent common ancestor with pattern 3, from which it diverged only near the 3′ extremity of the genome (Figure 2, panel B). Similarly, the genomes of patterns 7 and 8 were closely related from the 2B region through the middle of the 3C region and substantially diverged downstream. Genomic mosaicism is a common trait found in enterovirus ecosystems because of frequent recombination exchanges between cocirculating enteroviruses, including the poliovirus vaccine strains. Thus, VDPVs generally feature genomes resulting from multiple recombination events (*6*). Three VP1 branches (A, B, and D) contained various recombinant patterns (Figure 2, panel A); reciprocally, 2 recombinant patterns (3 and 11) were each found in several VP1 branches (Figure 2, panel B), thereby illustrating how recombination can make different segments of the enterovirus genome evolve independently (*7*).

Although VDPV-2s commonly harbor a recombinant nonpolio 5′ untranslated region (UTR), all CAR VDPVs had a 5′ UTR from the vaccine Sabin-2 strain. Nonetheless, an A→G reversion was found in all genomes at nt position 481, which harbors one of the major determinants of attenuation of the Sabin-2 strain (*8*). A second major determinant of Sabin-2, located within the VP1-encoding region (nt position 2909), had also reverted (U→C, isoleucine-to-threonine) in all CAR VDPVs.

## Conclusions

The origin of the CAR VDPV-2s remains unknown. In April 2016, a switch from use of the trivalent OPV to the bivalent OPV, which contains the Sabin-1 and Sabin-3 attenuated strains (but not Sabin-2), was synchronized globally (*9*). The low nucleotide divergence observed within the VP1-encoding sequence between the CAR VDPV-2s and Sabin-2 makes the hypothesis of silent circulation of Sabin-2–derived strains originating from the trivalent OPV over >3 years unlikely. More likely, the CAR VDPV-2s may derive from the Sabin-2 strain contained in the monovalent OPV that was used to control a 2017–2018 VDPV-2 outbreak in the Democratic Republic of the Congo, which borders CAR (*10*). Population movements across the border between the 2 countries could have allowed introduction of Sabin-2–derived viruses into CAR, a country in which most children born after the global vaccine switch have no immunity against serotype 2. The silent circulation of these viruses for several months was probably rendered possible by the difficulties of implementing efficient surveillance in some regions of CAR because of the civil war that has been ongoing in the country since 2012.

We show that the CAR VDPV-2 outbreak resulted from several independent emergence events, involving recombinant genomes with no recombination in the 5′ UTR. Beyond the situation in CAR, 2019 was a dark year for the Global Polio Eradication Initiative; VDPV-2 outbreaks surged in many countries in Africa (*11*). OPV of serotype 2 remains the best tool to stop VDPV-2 outbreaks, but it also constitutes the seed for emergence of VDPVs. The pending release of a novel OPV that contains a genetically stabilized serotype 2 strain less prone to reversion is expected to put an end to this vicious cycle (*12*).

Appendix Phylogenetic tree for type 2 vaccine-derived polioviruses, Central African Republic, 2019.
