# Supplementary material for: Vaccine-Derived Polioviruses, Central African Republic, 2019
Source: Emerg Infect Dis. 2021 Feb;27(2):620–3. doi: 10.3201/eid2702.203173 (PMC7853572; doi:10.3201/eid2702.203173)
Supplement: Appendix — Phylogenetic tree for type 2 vaccine-derived polioviruses, Central African Republic, 2019. [file 20-3173-Techapp-s1.pdf]

Article DOI: https://doi.org/10.3201/eid2702.203173

Vaccine-Derived Polioviruses, Central African Republic, 2019

Appendix

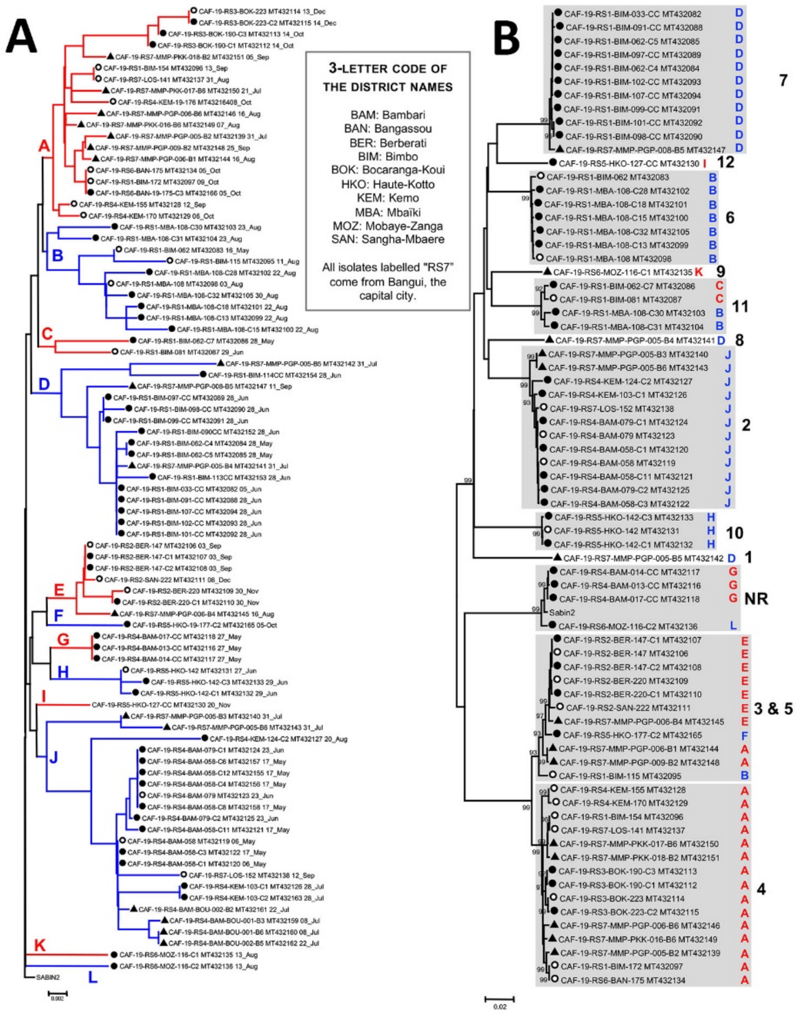

Appendix Figure. Phylogenetic relationships between VDPVs in the A) VP1 and B) 3D-encoding regions. Isolates from AFP cases are indicated by open circles, isolates from healthy children by black circles and isolates from environmental samples by triangles. The trees feature the full names of the isolates (with the district where they were sampled) and their respective GenBank accession number; the collection dates (all in 2019) are also indicated in the VP1 tree. The VP1-based tree is similar to that shown as Figure 2A. The 3D-based tree (Neighbor-Joining method with bootstrap values shown when >90%) highlights the recombinant patterns (1 to 12 and NR) shown in Figure 2B (patterns 3 and 5 are indistinguishable from each other in the 3D-encoding region, see Figure 2C); near each isolate name, the VP1 branch it belongs to (A to L) is indicated. Scale bars indicate nucleotide substitutions per site.
